# Safety and Efficiency of Low-Dose Spinal Analgesia Compared to Epidural Analgesia in Treatment of Pain during Labour: A Case Control Study

**DOI:** 10.3390/jcm12185770

**Published:** 2023-09-05

**Authors:** Martin Calineata, Lukas Jennewein, Vanessa Neef, Armin Niklas Flinspach, Frank Louwen, Kai Zacharowski, Florian Jürgen Raimann

**Affiliations:** 1 Goethe University Frankfurt, University Hospital, Department of Anaesthesiology, Intensive Care Medicine and Pain Therapy, Theodor-Stern-Kai 7, 60590 Frankfurt am Main, Germany; 2 Goethe University Frankfurt, University Hospital, Department of Gynaecology and Obstetrics, Theodor-Stern-Kai 7, 60590 Frankfurt am Main, Germany

**Keywords:** spinal anaesthesia, birth, epidural catheter, pain

## Abstract

Background: The epidural catheter for analgesia has been used for decades and has become the gold standard in pain therapy for pregnant women in labour. However, procedural parameters such as time to pain relief and duration to implementation pose hurdles for patients shortly before delivery. Low-dose spinal analgesia (LDSA) is an alternative procedure that was investigated in the study with regard to patient satisfaction and complication rates compared to epidural catheter. Methods: In a retrospective monocentric study, a total of 242 patients receiving low-dose spinal analgesia or epidural catheters were evaluated using propensity score matching. Subjective patient satisfaction as well as complication rates were primarily analysed. We hypothesise that LDSA is a safe procedure and provides a similar level of satisfaction compared with the epidural catheter. For this purpose, both procedures were performed according to in-house standards and the patients were interviewed afterwards. Patients who required surgical delivery were excluded to prevent bias. Results: The LDSA was rated on average as very good [1.09 ± 0.311 vs. 1.07 ± 0.431] in terms of satisfaction by the patients compared to the epidural catheter without showing a significant difference (*p* = 0.653). Complications were in the low single-digit non-significant range for both procedures [6 (5%) vs. 7 (6%); *p* = 0.776]. The evaluation showed more perineal tears I° and II° in the low-dose spinal analgesia group [I°: 28 (23%) vs. 3 (2%); *p* < 0.001—II°: 30 (25%) vs. 2 (2%); *p* < 0.001]. Neonatal parameters differed significantly only in umbilical cord base excess and umbilical cord venous pH [−5.40 vs. −6.40; *p* = 0.005]. Conclusions: LDSA represents a low complication procedure for patients at the end of labour with a high satisfaction level. With the LDSA in the repertoire of pain relief during childbirth, it is possible to also achieve pain reduction for women with deliveries of high velocity without compromising patient satisfaction or perinatal morbidity.

## 1. Introduction

Neuroaxial anaesthetic procedures are the most commonly used techniques for pain reduction during childbirth [1,2,3]. Over the last decades, different techniques such as epidural anaesthesia, spinal anaesthesia, or combined spinal epidural anaesthesia (CSE) as well as various drugs and dosages have been investigated [4,5,6]. Currently, epidural anaesthesia represents the gold standard and is one of the most commonly used procedures for pain reduction in pregnant women during childbirth [7]. However, all of these interventions may also have disadvantages, for instance, in the risk profile, but also in the duration until the onset of pain reducing effects. Particularly in pregnant women who are at the second stage of labour, and thus immediately prior to birth, the duration until the onset of pain reduction plays a central role. Multiparous women with naturally shorter delivery duration compared to nulliparous women need a rapidly efficient pain reduction above all.

For technical reasons, the time between the insertion of the introducer needle and the onset of the effect of epidural anaesthesia is approximately 30 min. In order to achieve a faster onset of pain reduction, higher doses are necessary, but this involves an increased risk of motor blockade [8]. Although other techniques such as epidural electrical stimulation exist to determine the correct location, the administration of a test dose remains a widely used technique [9]. In order to provide rapid pain relief to pregnant women at the end of birth, who have not yet received a pain reduction procedure, there is the possibility of so-called “low-dose spinal analgesia (LDSA)” (syn: “rescue spinal analgesia”) [10], as recommended by the German guidelines for breech at term and obstetric analgesia and anaesthesia [11,12,13]. Intrathecal administration results in a rapid onset of pain reduction. In contrast to an epidural catheter, the administration of a test dose can be dispensed with. Therefore, this less common procedure is particularly suitable at a late stage of birth or for multiparous women.

We therefore conducted a retrospective monocentric study and evaluated the LDSAs performed at our institution with a primary focus on patient satisfaction. Additional secondary outcome parameters were birth related injury, need for additional anaesthetic procedure, Apgar, arterial and venous umbilical cord pH, umbilical cord base, and length of stay (hospital and intensive care unit for the parturient and newborn).

## 2. Materials and Methods

This retrospective, observational, case-control, monocentric study was conducted at the University Hospital Frankfurt, Goethe University, Frankfurt, Germany in cooperation between the Department of Anaesthesiology, Intensive Care Medicine, and Pain Therapy and the Department of Gynaecology and Obstetrics.

It was performed according to the principles and actual version of the Declaration of Helsinki [14]. Approval was obtained from the local ethical committee of University Hospital Frankfurt on 5 May 2021 (Identifier: 2021-176) and written consent was not required.

### 2.1. Study Design

Digital patient records for the evaluation period 1 September 2012 to 1 November 2020 were scanned from the study team for eligible patients who met the inclusion criteria. The inclusion criterion was defined as having received a LDSA as well as patients receiving an epidural anaesthesia during the second stage of labour. The exclusion criteria were defined as having a secondary caesarean section (sCS), stillbirth, procedural failure, or the need for an additional anaesthetic procedure, and combined spinal-epidural anaesthesia (CSE). These exclusion parameters were used to avoid a bias based on mixed procedures and different pain experience by sCS.

Within this timeframe, two groups of patients were evaluated and named the LDSA and epidural analgesia (EA) groups.

The EA group included all patients who received a LDSA during childbirth at the University Hospital Frankfurt. To evaluate the satisfaction and periprocedural complications, a control group (=EA group) was defined as patients who received an epidural catheter whilst being in the second stage of labour. Every patient in the LDSA group was matched by propensity score matching with a corresponding patient in the EA group.

**Figure 1:**
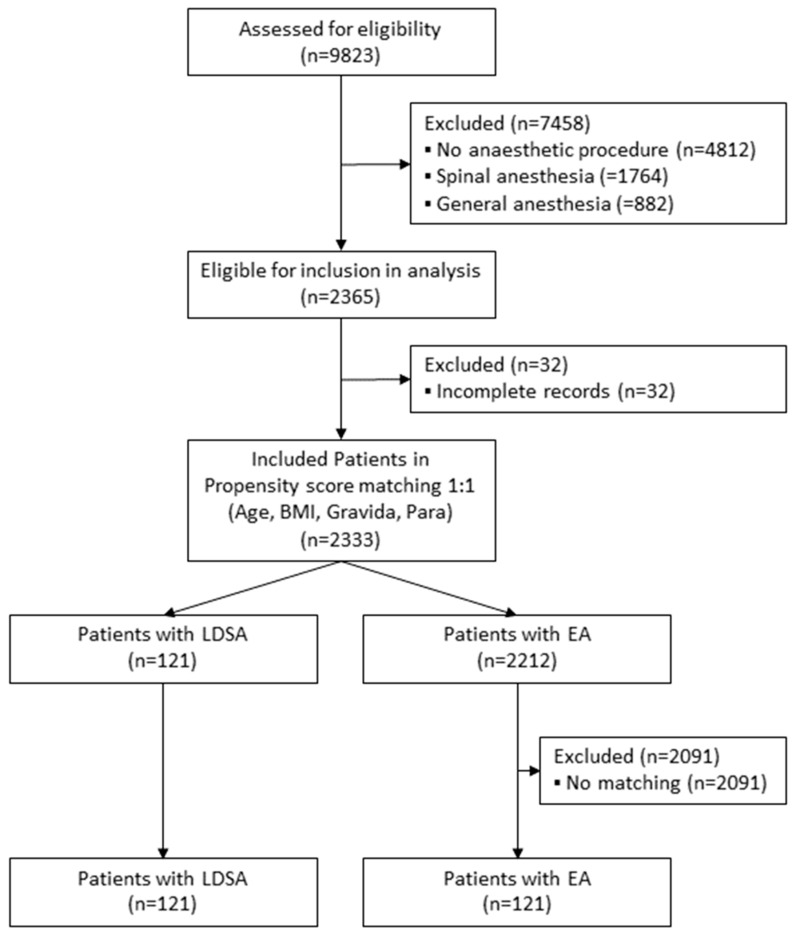
Flowchart of the study design and propensity score matching. BMI = body mass index (kg/m^2^); LDSA = low-dose spinal analgesia; EA = epidural analgesia.

The analysed and excluded number of patients including the reasons for exclusion are depicted in the consort flow diagram (Figure 1).

### 2.2. Data Collection and Preparation

As of 1 September 2012, all anaesthesiology services performed are recorded electronically. Since then, the visits of the anaesthesiological rounds have also been documented in the patient data management system (PDMS). To evaluate a maximum of datasets, the start of the evaluation period was set to this date.

Data were extracted from two institutional PDMSs named Orbis (Agfa HealthCare, Mortsel, Belgium) and GeDoWin (Saatmann GmbH, Worms, Germany).

The data collected comprised the demographics, clinical characteristics, information on labour (number of pregnancies and deliveries of a mother giving birth, singleton or multiple pregnancy, foetal birthweight, foetal birth length, birth related injuries, need for further anaesthesiologic procedures, cord pH and cord base excess, Apgar-score [15], length of stay (LOS) as well as information on the performed procedure (date and time, qualification of the anaesthetist in charge, patient satisfaction (ranked 1–6), number of punctions (=number of needle insertions independently from vertebral level), complications). All data were anonymized.

### 2.3. Outcome Parameters

The primary outcome was to assess the patient satisfaction according to the Likert scale (grades 1 (very good) to 6 (very poor) as collected by an anaesthetist during visitation the day after birth; at least after 12 h postpartum).

Secondary outcome parameters focused on the safety for the parturient and foetus. For the parturient, we compared the rate of birth injury, the necessity of other forms of anaesthesia (spinal anaesthesia, general anaesthesia for caeasarean section, or epidural anaesthesia for caesarean section). The safety for the foetus was assessed using foetal cord pH (venous and arterial) and base, Apgar-score at one, five, and ten minutes after delivery, admission to neonatal intensive care unit (NICU), time spent on the NICU, and overall length of stay (LOS).

### 2.4. LDSA and Epidural Anaesthesia

The institutional standard of care according to the local standard operation procedures (SOP) for the second stage of labour includes a LDSA for parturients in advanced stage of labour, as referred to by guidelines from the German Society of Anaesthesiology [11]. In our clinic, LDSA is used under strict criteria and is applied to patients at the endstage of delivery. In some cases, LDSA is applied to patients who have an indication for instrumental delivery (e.g., non-reassuring foetal heart rate pattern or arrest in stage II of labour) and do not have established analgesia at this point. Instrumental deliveries are performed in our clinic by forceps and not by vacuum extraction. Then—in order to apply sufficient analgesia quickly—LDSA is recommended by the obstetrician.

Standard monitoring (non-invasive blood pressure, peripheral oxygen saturation and electrocardiography) was established on arrival of the attending anaesthesiologist in the delivery room.

In the LDSA group, spinal analgesia was performed after skin disinfection (3× with octeniderm (Schülke & Mayr GmbH, Norderstedt, Germany) over a time period of at least 3–5 min. After drying, a sterile cover was placed over the site of puncture, followed by the injection of 5 mL 1% Mepivacaine (Scandicain^®^, Aspen, Munich Germany) as the local anaesthetic under sterile conditions.

After a sufficient level of anaesthesia was reached, a 25 G (0.53 × 88.00 mm) Pencan^®^ Pencil Point needle (B. Braun, Melsungen, Germany) was inserted. After the identification of spinal space with leakage of cerebrospinal fluid, a mixture of 4–5 mg (≜2.0–2.5 mL) Ropivacaine 0.2% (Naropin^®^, Aspen, Munich, Germany) and 5 μg (≜1 mL) Sufentanil (Fa. Hameln, Hameln, Germany) were injected.

In the EA group, local anaesthesia was performed with 5 mL Mepivacaine, skin disinfection, and sterile covering. After aa sufficient level of anaesthesia was reached, a Tuohy-needle (Vygon GmBH, Aachen, Germany) was inserted using the loss-of-resistance method to identify the epidural space. After identification, a small plastic catheter was inserted and the skin level documented. Thereafter, a test dose of 5 mL Ropivacaine 0.2% was injected. Within 10 min after administration of the initial Ropivacaine dose, the blood pressure and sensorimotor state were examined. In the case of absent effects, an additional 8 mL of Ropivacaine 0.2% (=16 mg) and 10 μg of Sufentanil epidural were injected. The catheter was fixed by Steri-Strip™ (3M Medica, Neuss, Germany) and sealed with a plaster.

All parturients, independent from the procedure, were monitored a minimum of 30 min after neuroaxial intervention. If there was no evidence of adverse hemodynamic events and the pain relief was sufficient, the parturients were observed by the obstetrician in charge.

### 2.5. Statistical Analysis

Statistical analyses were performed with the support of the Institute of Biostatistics (University Hospital Frankfurt, House 11, Theodor-Stern-Kai 7, 60590 Frankfurt am Main, Germany). The data were analysed using SigmaPlot version 12 (Systat Software, Erkrath, Germany) and IBM SPSS Statistics (IBM Corp. Released 2020. IBM SPSS Statistics, Version 22.0., Armonk, NY, USA). After checking for eligibility and overall analysis, propensity score matching (Calipher 0.2) was used (used parameters for matching: body mass index (BMI); age; gravida and para) to generate an equally sized control group that received an epidural catheter (Figure 1).

Depending on the distribution and scale of data (determined via the Shapiro–Wilk test), the paired *t*-test, Wilcoxon test, or Mann–Whitney rank sum test were used to compare the data. The chi^2^ test was used to detect differences between the proportions of patients with respect to the categorical data. For multi-variable analysis, a logistic regression model was used. Values were expressed as the number (percentage), mean ± standard deviation, or median (IQR, interquartile range), as appropriate. All tests were two-sided and a *p* value of less than 0.05 was considered to be statistically significant.

## 3. Results

**Figure 2:**
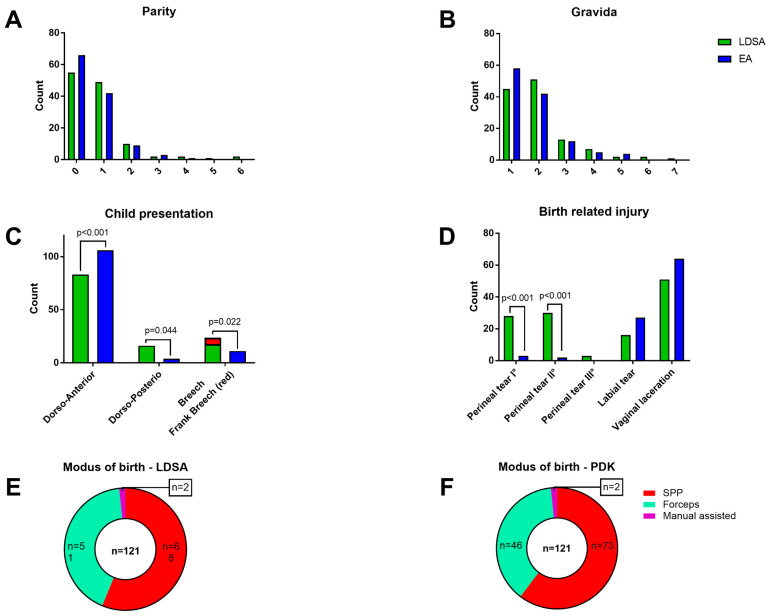
Birth related data. LDSA = low-dose spinal analgesia; EA = epidural analgesia; (**A**) Parity; (**B**) Gravida; (**C**) Child presentation: Frank breech presentation (LDSA *n* = 5; EA *n* = 0; *p* = 0.022) is displayed as part of overall breech presentation (red square); (**D**) Birth related injury, (**E**,**F**) Modus of birth for LDSA and EA groups, SPP = spontaneous partus.

In total, 242 patients giving birth to 244 newborns were evaluated: 121 patients in the LDSA group and 121 in the EA group. There was no significant difference in the age, weight, height, or BMI detected (Table 1). Figure 2A–F depicts the obstetric characteristics of the two groups. Figure 2A shows parity (1 to 6), Figure 2B gravida (1 to 7), Figure 2C the kind of child presentation (Frank breech presentation as part of breech presentation was only detected in LDSA group (red square)), Figure 2D the birth related injury, and Figure 2E,F the modus of birth for LDSA, respectively, for the EA group.

Table 2 shows the newborn parameters as well as the NICU admissions and length of (hospital) stay for mothers and newborns.

**Figure 3:**
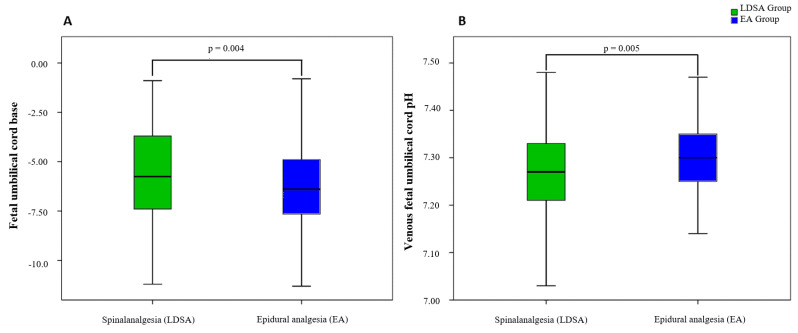
Umbilical cord blood gas analysis. LDSA = low-dose spinal analgesia; EA = epidural analgesia; (**A**) Fetal umbilical cord base; (**B**) Venous fetal umbilical cord pH.

In total, 21 parameters were evaluated. Of these 21 parameters, seven reached the level of significance (child presentation, birth related injury, and umbilical cord blood gas analysis) (Figure 2C,D; Figure 3; Table 2). The Frank breech presentation (red square) are displayed as part of the overall breech presentation. The LDSA group contained five frank breech presentations compared to zero in the EA group (*p* = 0.022). The overall breech presentations did not differ significantly between both groups.

The demographic data (Table 1) did not differ between both groups, neither did the mode of birth (Figure 2E,F), Apgar-score, length of stay (LOS) for mothers or newborns, neonatal intensive care unit (NICU) admissions, or newborn seize and weight (Table 2).

**Figure 4:**
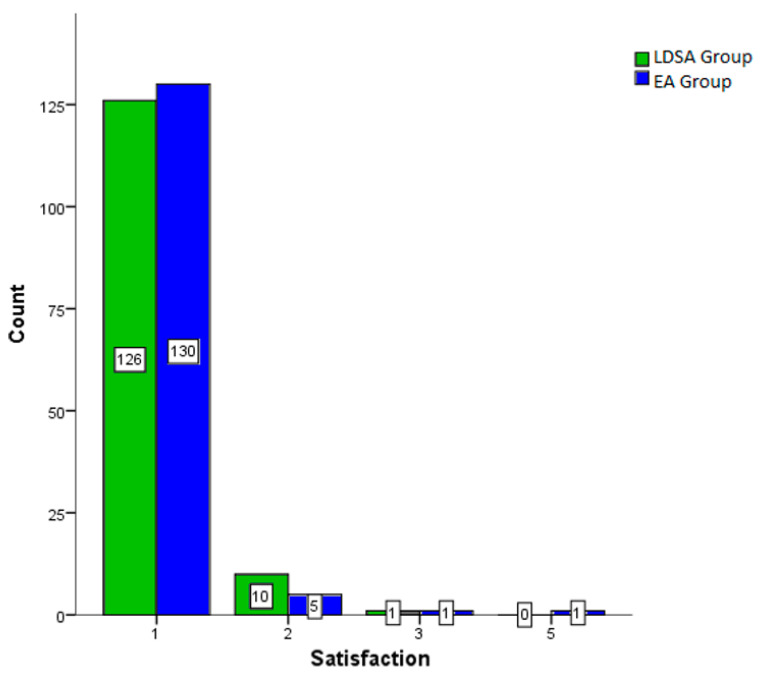
Satisfaction. LDSA = low-dose spinal analgesia; EA = epidural catheter; Grade 1 = very satisfied to Grade 6 = very unsatisfied. Each column shows the absolute count in squares.

Figure 4 shows the satisfaction (Grade 1–6; 1 = very satisfied; 6 = very unsatisfied). There was no significant difference between both groups (*p* = 0.653).

Looking at the satisfaction in dependence of the qualification of the performing anaesthetist, there was no significant difference to detect. Whether a senior physician, a resident, or a trainee performed the spinal analgesia or the epidural catheter did not influence the satisfaction scores (*p* = 0.357).

There was no significant difference between both groups regarding the qualification of the performing anaesthetist in charge (Table 3).

When evaluating the complications that occurred in both groups, there was no significant difference (*p* = 0.776). In the LDSA group, three post-puncture headaches, two bone contacts with new needle insertion, and one maternal bradycardia occurred, while in the EA group, three epidural catheter replacements, one bone contact, one catheter dislocation, one post-puncture headache, and one hematoma at the punction side were noted.

The qualification of the performing anaesthetist had no influence on the occurrence of complications (*p* = 0.290).

The number of punctions did not differ significantly between both groups with one punction in median (*p* = 0.113). The qualification of the anaesthetist in charge had no influence on the number of punctions (*p* = 0.174). 

Table 3 shows the results of the analysis of the performed anaesthetic procedures such as satisfaction, bloody tap, qualifications, number of punctions, and occurrence of complications as well as the performed modus of delivery (Figure 2E,F).

Looking at birth related injuries, significantly more first and second-degree perineal lacerations were noted in the LDSA group, while all other types of injury (third-degree perineal tear, labial injury, or vaginal injury) did not differ. The overall count of birth related injury was 92 vs. 101 in the LDSA vs. EA group (*p* = 0.926). A total of 76% of all patients in the LDSA group and 83% in the EA group suffered from birth-related injuries. None of these women required an additional anaesthetic procedure for an ongoing intervention. LDSA or EA were adequate for surgical care in each case.

Foetal umbilical blood gas analysis (BGA) showed a significant difference in venous BGA with a reduced pH value in the LDSA group (7.27 vs. 7.30; *p* = 0.004; Figure 3B), while there was no difference detected in the arterial BGA. Foetal umbilical base, measured in the arterial BGA, showed a lower value in the LDSA group (−5.40 vs. −6.40; *p* = 0.005; Figure 3A).

## 4. Discussion

LDSA is a low-complication procedure for patients at the end of labour with a high degree of satisfaction. Patient satisfaction was shown to be high, with no difference in group comparison. Relevant neonatal parameters showed no group differences, suggesting that LDSA is not a relevant risk factor for neonates. 

Therefore, especially in deliveries of high velocity, LDSA might be beneficial compared to an epidural catheter analgesia because the time interval between application and the onset of pain relief is much shorter. Therefore, LDSA is a less common but effective procedure to provide pain relief to pregnant women in the final stage of labour [16,17,18,19,20].

Besides the rapid onset of analgesic effects, it must be taken into account that all neuraxial labour analgesia may prolong the second stage of labour. LDSA may be beneficial in the case of the need for an assisted birth. Intrapartum ultrasound seems to be an adequate option to determine the foetal head progression [21]. Due to the fact that the patients included in this study were about to give birth immediately, patients in whom CSE was an option were not included. Identification of the epidural space and catheter placement would have required a time delay. The goal was to allow a return to a birthing position as soon as possible in patients in labour. Due to the low prevalence of this anaesthetic procedure, only a few studies that mostly considered a few participants exist to date [16,17,18,19,20,22]. Based on different study designs as well as inclusion and exclusion criteria, comparability is extremely limited, making it difficult to draw conclusions about patient satisfaction, effectiveness, and safety.

In obstetric anaesthesia, the epidural catheter represents the gold standard and is much more widespread than the LDSA [22,23,24]. Considering patient overall satisfaction as the primary endpoint, we could show that patients who received LDSA were highly satisfied with the pain reduction and the procedure in general. Thus, patients rated it a mean score of 1.1 (±0.311) on the Likert scale (1 = very satisfied; 6 = very dissatisfied). To compare this rating with the gold standard EA, we used propensity score matching to assess satisfaction in a comparison group (EA group). Here, the EA procedure was also rated with a mean score of 1.1 (±0.431). These results are comparable to a study by Rahmati et al., who also found excellent pain reduction in a group of 128 patients (*n* = 64 LDSA; *n* = 64 EA). It must be mentioned that the number of patients was smaller than in our study and that different dosages were used for the LDSA. In addition, Rahmati’s study included patients who underwent a secondary caesarean section [22]. This leads to a bias with regard to postoperative pain development. For this reason, patients who received a surgical delivery were excluded in our investigation. In contrast, this allowed us to obtain a more accurate picture of the particular procedure chosen on pain development and patient satisfaction. Viitanen et al. investigated, without a EA comparison group, 209 multiparous women who received LDSA using bupivacaine and fentanyl. However, this study did not exclude patients who received surgical delivery, which may have biased the data. Nevertheless, 153 (73%) reported satisfactory pain reduction, although this study did not explicitly ask about their satisfaction with the LDSA procedure. Nevertheless, Viitanen’s study showed that the time from application to pain reduction was achieved after only a few contractions [20]. In another study evaluating LDSA by Kuczkowski et al., a total of 62 female patients were examined with regard to satisfaction. In this study, a triple combination (bupivacaine, morphine, and clonidine) was used. Due to the use of clonidine for effect prolongation and morphine, which is not recommended for this indication in Germany, a direct comparison with our data is difficult. Additionally, Kuczkowski et al. lacked a EA comparison group. Nonetheless, this study also showed high patient satisfaction with 81% very satisfied patients and another 11% who were satisfied [17].

Different recommendations are given for pregnant women with unborn in breech presentations. Our obstetric clinic specialises in vaginal delivery in breech presentation. An EA as well as LDSA are part of the standard clinical treatment and is offered to every woman giving birth. During pregnancy counselling in breech presentation, women are informed about the possible unknown effects of an epidural anaesthesia on the course of birth. Our overall caesarean section rate in deliveries with breech presentation is about 30%, as can be seen in our publication of the FRABAT study collective [25]. In recent years, we have recorded high patient satisfaction and a low complication rate, which is why this procedure is now standard in the obstetrics department. 

Looking at the drug doses chosen, Tshibuyl et al. examined different dosing regimens for single injection analgesia. Here, two groups (fentanyl + bupivacaine or fentanyl + bupivacaine + morphine) were compared, while we used ropivacaine + sufentanil. As expected, the additive use of morphine showed a prolongation of effect and correspondingly higher patient satisfaction [19]. Critically, the increased side effect profile due to the additional administration of morphine must be considered in this case. For example, intrathecal administration of morphine for caesarean section leads to a decrease in satisfaction and episodes of hypercapnia [26,27]. Late respiratory depression occurs after predominantly 5 h and mainly at night. Thus, there is no recommendation to use morphine additively in the current national guideline on obstetric analgesia and anaesthesia, which is also reflected in our chosen drug regimen [11].

Considering the maternal complication rate of LDSA (*n* = 6; 5%) compared to EA (*n* = 7; 6%), there was no significant difference in our investigation (*p* = 0.776). Known side effects such as pruritus, which are sufficiently known due to the opioid component, were not taken into account. Kuczkowski et al. and Rahmati et al. did not detect any relevant complications either [17,22]. Viitanen et al. described five (=2%) cases of hypotension in their studied patients [20]. Eriksson et al., who studied 40 patients receiving intrathecal single-shot analgesia, detected six (=15%) cases of hypotension [16]. We, on the other hand, did not experience any haemodynamic instability. This difference may be due to different drug regimens and different applied volumes. Only Sharpe et al. found nine (2.1%) cases of postdural puncture headache (PDPH) in a study on the necessity of adding analgesics after single injection spinal analgesia. However, no EA group to compare was evaluated in Sharpe’s study. Sharpe et al. believes that the causes of PDPH are multiple punctures and the use of a Whitcare needle in some cases. Looking at the number of PDPH, in our study, in the group of LDSA patients (*n* = 3; 2.5%), it was in the same range as in Sharpe’s investigation [18].

Virtually ignored in the comparative studies is the question of birth injury. Only Eriksson et al. reported two cases of anal sphincter tears (=5%) [16]. To the best of our knowledge, we are the first to investigate birth-related injuries in terms of LDSA compared to EA as an anaesthetic procedure. Figure 1 shows the detailed breakdown of birth-related injuries depending on the anaesthetic procedure chosen. Thus, we were able to show that significantly more perineal tears I° and II° were present in the LDSA group. A potential explanation for this could be a higher number of infants with a dorsoposterior presentation in the LDSA group. However, it could also be the reduced pain sensation shortly before delivery, which may lead to a more expeditious delivery. However, a comparison with other studies is not possible at this time.

In our study, the neonatal parameters of Apgar-score and umbilical cord pH showed significant differences only in the range of umbilical cord base excess and venous umbilical cord pH (Table 2). Looking at the Apgar-score, our results are in line with those of Eriksson et al., although there was no EA comparison group [16]. Only Rahmati et al. resorted to a comparison group. Here, a trend towards better Apgar-score at the time of 1 min was found without reaching the level of significance. The Apgar-score at the 5-min time point was consistent with our results [22]. However, no data on the Apgar-score at 10 min can be found in the literature. The greater relevance in neonatal assessment is represented by the arterial umbilical pH compared with the venous. In an analysis by Yeh et al., the threshold for the occurrence of severe complications was set at pH 7.10, which we did not fall below in any case in our investigation [28]. Admission to the neonatal intensive care unit (NICU) also did not differ between the two groups we studied. Unfortunately, there is no comparative value in the literature at the present time for this studied parameter, so we are the first to account for it.

### Limitations

Based on the retrospective design of this study, the data may be of lower quality compared to prospective studies. The low number of patients investigated reduces the power of the conclusions drawn from our results. More detailed prospective studies based on this topic are necessary to gain a deeper insight into LDSA.

## 5. Conclusions

LDSA represents a low-complication procedure for patients at the end of labour with a high level of satisfaction, although no difference was seen in terms of satisfaction compared with patients who received an EA. We were able to detect a higher rate of perineal tears I° and II°, which should be the subject of further investigation. The relevant neonatal parameters showed no group differences, suggesting that LDSA is not a relevant risk factor for the neonates.

## Figures and Tables

**Table 1:** Demographic and pregnancy data.

	LDSA (*n* = 121)	EA (*n* = 121)	Overall (*n* = 242)	*p*-Value
Age [Year]	33.0 (30.0/35.0)	33.0 (30.0/35.0)	33.0 (30.0/35.0)	0.390 ^&^
Weight [kg]	73.0 (66.0/82.5)	72.0 (66.0/82.0)	73.0 (66.0/82.0)	0.909 ^&^
Height [cm]	167.4 (±6.7)	167.3 (±6.3)	167.1 (±6.5)	0.999 ^&^
BMI [kg/m^2^]	26.0 (24.1/29.0)	26.0 (24.1/29.0)	26.0 (24.1/29.0)	0.452 ^&^
Singleton [*n*, %]	120 (99%)	120 (99%)	240 (99%)	1.000 ^§^
Gemini [*n*, %]	1 (1%)	1 (1%)	2 (1%)	1.000 ^§^

BMI = body mass index, median (interquartile range (IQR) 25/75); LDSA = low-dose spinal analgesia; EA = epidural anaesthesia; *n* = number; % = percentage; age; weight; height: mean ± standard deviation; interquartile range; ^§^ = chi-square test; ^&^ = Wilcoxon test.

**Table 2:** Newborn related data.

	LDSA (*n* = 121)	EA (*n* = 121)	Overall (*n* = 242)	*p*-Value
Arterial umbilical cord pH	7.21 (±0.08)	7.21 (±0.07)	7.21 (±0.08)	0.483 ^$^
Venous umbilical cord pH	7.27 (±0.09)	7.30 (±0.08)	7.28 (±0.08)	0.004 *^$^
Foetal umbilical base	−5.40 (7.70/−3.40)	−6.40 (−8.00/−4.90)	−5.95 (−7.80/−4.30)	0.005 *^#^
Apgar 1	9 (9/9)	9 (9/9)	9 (9/9)	0.766 ^#^
Apgar 5	10 (10/10)	10 (10/10)	10 (10/10)	0.701 ^#^
Apgar 10	10 (10/10)	10 (10/10)	10 (10/10)	0.212 ^#^
Newborn Weight [g]	3390 (3080/3680)	3480 (3160/3815)	3400 (3040/3725)	0.141 ^#^
Newborn Size [cm]	51 (50/53)	51 (50/53)	51 (50/53)	0.878 ^#^
NICU-Admission [*n*, %]	15 (12%)	11 (9%)	26 (11%)	0.541 ^$^
LOS Newborn [dd:hh:mm]	08:01:22 (07:00:22/12:01:30)	08:03:58 (06:07:55/10:02:42)	08:02:20 (07:00:13/11:00:58)	0.241 ^&^
LOS Mother [dd:hh:mm]	09:07:15 (07:06:57/13:00:09)	10:07:54 (09:01:28/15:03:23)	10:04:18 (08:02:00/14:03:18)	0.267 ^&^

NICU = newborn intensive care station; LOS = length of stay; LDSA = low-dose spinal analgesia; EA = epidural anaesthesia; *n* = number; % = percentage; arterial umbilical cord pH; venous umbilical cord pH; foetal umbilical base: mean ± standard deviation; Apgar 1,5,10; newborn weight + size, LOS newborn + mother: median (interquartile range (IQR) 25/75); ^#^ = U-test; ^$^ = paired *t*-test; ^&^ = Wilcoxon test; * = significant result.

**Table 3:** Anaesthetic procedure details and patient satisfaction.

	LDSA (*n* = 121)	EA (*n* = 121)	Overall (*n* = 242)	*p*-Value
Satisfaction [Grade 1–6]	1 (1/1)	1 (1/1)	1 (1/1)	0.653 ^#^
Bloody tap [*n*, %]	0 (0%)	2 (2%)	2 (1%)	0.154 ^$^
Qualification [*n*, %]				
Senior physician	9 (7%)	4 (3%)	13 (5%)	0.152 ^$^
Resident	6 (5%)	10 (8%)	16 (7%)	0.300 ^$^
Trainee	106 (88%)	107 (88%)	213 (88%)	0.579 ^$^
Needle insertions	1 (1/1)	1 (1/1)	1 (1/1)	0.113 ^#^
Complications anaesthetic procedure [*n*, %]	6 (5%)	7 (6%)	13 (5%)	0.776 ^$^

LDSA = low-dose spinal analgesia; EA = epidural anaesthesia; satisfaction grading: 1 = poor to 6 = very good; satisfaction; needle insertions: median (IQR 25/75); *n* = number; % = percentage; ^#^ = U-test; ^$^ = paired *t*-test.

## Data Availability

Data can be obtained from the corresponding author.
